# Estimating Changes in Population Size and Behavioral Characteristics in Men Who Have Sex With Men Between 2014 and 2019: Longitudinal Study

**DOI:** 10.2196/34150

**Published:** 2022-08-16

**Authors:** Zijie Yang, Lan Wei, Wei Xie, Lin Chen, Zhengrong Yang, Yan Zhang, Shaochu Liu, Wei Tan, Chenli Zheng, Yongxia Gan, Dongmin Li, Huachun Zou, Wanying Chen, Ling Ma, Niu Ju, Yinghui Sun, Fan Lv, Jin Zhao

**Affiliations:** 1 Shenzhen Center for Disease Control and Prevention Shenzhen China; 2 School of Public Health Peking Unversity Beijing China; 3 School of Public Health Shantou Unversity Shantou China; 4 National Center for AIDS/Sexually Transmitted Disease Control and Prevention Chinese Center for Disease Control and Prevention Beijing China; 5 School of Public Health Sun Yat-sen University Shenzhen China; 6 Binhu District Centers for Disease Control and Prevention Wuxi China

**Keywords:** men who have sex with men, population size, HIV/AIDS, behavioral characteristics

## Abstract

**Background:**

Men who have sex with men (MSM) are at high risk for HIV infection. Accurate estimation of the population size and monitoring the risk sexual behavioral change of MSM is of great importance to develop targeted HIV prevention and interventions.

**Objective:**

The goal of the research was accurate estimation of the population size and monitoring the risk sexual behavioral change of MSM.

**Methods:**

Street interception investigation methods were conducted among males aged 16 years and older in selected sites in Shenzhen in 2014 and 2019. A population survey was used to estimate the population size of MSM. Logistic regression analysis was applied to evaluate the difference in behavioral characteristics in MSM from 2014 to 2019.

**Results:**

In this study, we surveyed 10,170 participants in 2014, of whom 448 (4.41%, 95% CI 4.01%-4.80%) participants were men who have ever had sex with another man (MSMe) and 229 (2.25%, 95% CI 1.96%-2.54%) were men who had sex with another man in the previous 6 months (MSMa). A total of 10,226 participants were surveyed in 2019, of which 500 (4.90%, 95% CI 4.47%-5.31%) and 208 (2.03%, 95% CI 1.76%-2.31%) participants were MSMe and MSMa, respectively. The results showed that the population size of MSM who are active (MSMa) in Shenzhen was 155,469 (2.29%, 95% CI 2.28%-2.30%) in 2014 and 167,337 (2.05%, 95% CI 2.04%-2.06%) in 2019. It was estimated that there were about 12,005,445 (2.04%, 95% CI 2.04%-2.04%) MSMa in China in 2019. Compared with 2014, the MSMa in 2019 were more likely to seek sex partners through mobile phone apps and less likely to have male and female sex partners in addition to having inconsistent condom use and more than 6 sex partners in the previous 6 months.

**Conclusions:**

In Shenzhen, the proportion of MSMa among the general male population was lower in 2019 than in 2014, and the prevalence of HIV risk behavior was reduced in 2019. Although the preferred platform to find male sex partners among MSM has changed, intervention with high–HIV risk MSM could still help to reduce HIV risk behaviors among the whole MSM group. Because MSM prefer to seek sex partners through mobile phone apps, further study is needed to strengthen internet interventions with high–HIV risk MSM to curb the spread of HIV.

## Introduction

Men who have sex with men (MSM) are at high risk of HIV infection [1]. Previous studies suggested that the prevalence rate of HIV among MSM increased from 0.9% in 2003 to 6.3% in 2019 in China [2,3], and the proportion of MSM among newly diagnosed HIV/AIDS cases increased from 13.7% in 2011 to 23.3% in 2019 [4,5]. In Shenzhen, of the 99.6% of newly diagnosed HIV/AIDS cases in 2020 infected through sexual transmission, 62.6%were infected through male-to-male sexual transmission [6]. Accurate estimation of the prevalence of HIV among MSM depends on the precise population size of MSM, which remains underinvestigated in China. Accurate estimation of the precise population size of MSM could help us assess our progress toward the World Health Organization HIV testing and condom use targets of 95% coverage by 2030 [7].

Guidelines released by the Joint United Nations Programme on HIV and AIDS and the World Health Organization on estimating the size of populations most at risk of HIV infection recommend several methods (census and enumeration, capture-recapture, and multiplier) for estimating the MSM population size using data collected from the population at risk in addition to methods based on the general population (eg, population survey, network scale-up) [8]. In China, previous studies have mostly used the capture-recapture [9], multiplier [10], and network scale-up methods [11] to estimate the size of the MSM population; population survey has never been reported. The capture-recapture and multiplier methods mostly collect data from MSM venues, MSM websites, and social media, which may introduce sample selection bias by ignoring MSM who seek sex partners through other ways. MSM completing online surveys are more likely to be socially and sexually active [12]. Using the network scale-up method to estimate the size of the MSM population may introduce transmission error [8], meaning participants may be unaware that some of their network members are MSM because most MSM tend to conceal their homosexual status from family, relatives, and friends due to the stigma against homosexuality [13,14]. All methods mentioned above may contribute to underestimation of the size of the MSM population.

In recent years, social media has changed the way of social networking among MSM [15], especially for their homosexual partners [16]. Data on sentinel surveillance showed that HIV prevalence of MSM in some Chinese metropolises has been stable or slightly declining in the past several years [17], which might be attributed to the persistent and intensive HIV intervention and education focusing on MSM [18]. However, previous surveillance and intervention were implemented in MSM venues or their social network. The same sampling sources might lead to poor representativeness and exaggerate the effects of intervention. Whether similar change in behavioral patterns could be observed in all MSM needs further investigation. In this study, we used population survey with a quantifiable sampling frame to screen MSM samples from the general population, which is more representative and reliable for estimating MSM population size than other methods [19].

Shenzhen, the first city of reform and opening up in China, has shown high acceptance of homosexual culture and diverse MSM social venues [20]. A previous study suggested that Shenzhen is one of the most popular gathering places for MSM in China, with about 90% of MSM in Shenzhen being a floating population from all over the country [21]. Accurate estimation of the population size of MSM and evaluation of their behavioral changes are imperative for effective decision-making on public health resources allocation and planning and management of HIV prevention programmed to MSM. Therefore, we conducted a population survey among MSM in Shenzhen with aims to estimate the population size of MSM in Shenzhen and examine the changes of HIV risk behavioral characteristics of MSM in 2014 and 2019.

## Methods

### Data Collection

**Figure 1:**
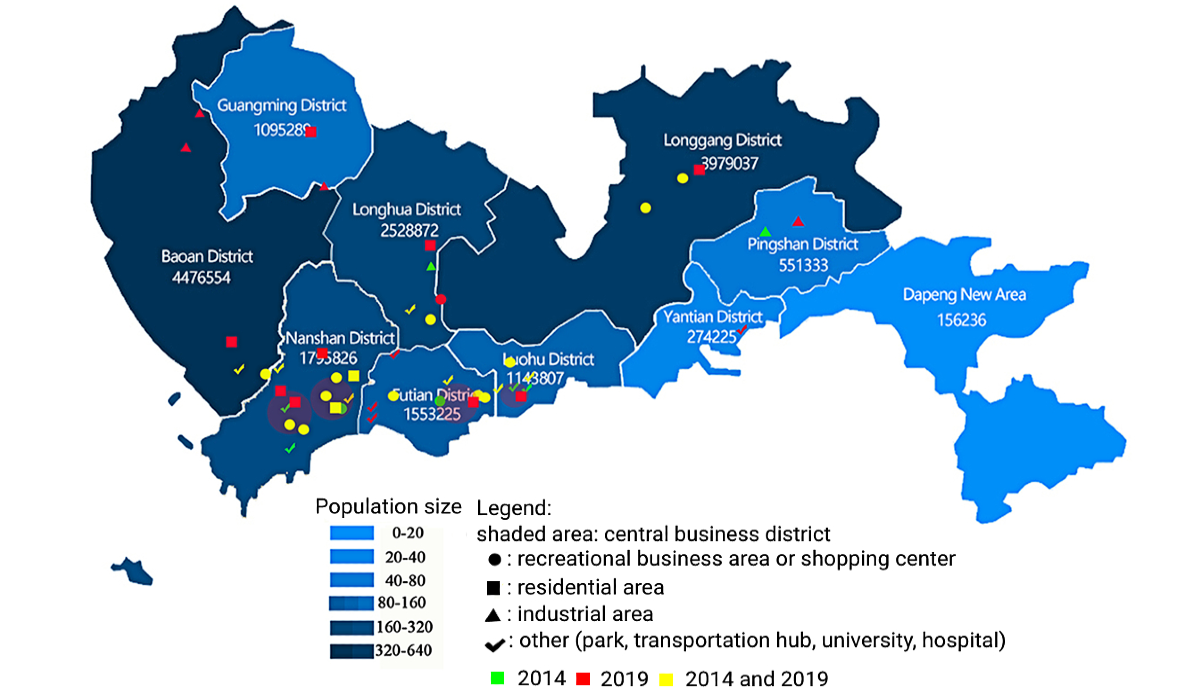
Distribution of investigation sites in Shenzhen.

We selected investigation sites in Shenzhen in 2014 and 2019 according to the population, business district distribution, and the personnel migration (Figure 1, Table 1). Sites in the vicinity of MSM social venues were excluded. A street interception investigation method was conducted among males aged 16 years and older in selected sites. We adjusted the age and spatial distribution of the sample according to the age and spatial distribution of the total male population.

Each participant was required to complete a self-administered questionnaire on the tablet computer or paper questionnaire in 2014 and in 2019 by tablet computer or online questionnaire by scanning a QR code at designated sites. Duplicate participation was rejected after automated phone number check. Participants were reimbursed with a random amount ranging from 5 to 100 RMB (US $0.80-$16.00) by scanning one-time QR code in WeChat Pay after completing the questionnaire. The questionnaire consists of 2 sections: sociodemographic characteristics and HIV risk behaviors. Sociodemographic characteristics include age, educational level, household registration, length of time staying in Shenzhen, marital status, sexual orientation, and gender of sex partners. Participants who answered the gender of sex partners question male or male and female were asked to complete the section about HIV risk behaviors, which included preference for ways of social networking, number of male sex partners in the previous 6 months, frequency of sex with men in the previous 6 months, frequency of condom use during sex with regular/nonregular male sex partners in the previous 6 months, history of sexually transmitted infections (STIs), and HIV testing in the previous year.

**Table 1:** Types of investigation sites in Shenzhen in 2014 and 2019.

Sites	2014, n	2019, n
Recreational business area or shopping center	22	16
Residential area	5	11
Park	10	7
Transportation hub	1	1
University	3	2
Hospital	3	3
Industrial area	4	5
Total	48	45

### Ethics Approval

This study was performed in line with the principles of the Declaration of Helsinki. Approval (SZCDC2019-010A) was granted by the ethics committee of the Shenzhen Center for Disease Control and Prevention.

### Calculation of Sample Size

The formula for calculating the sample size was computed as



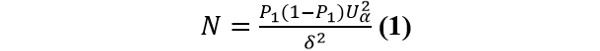



*α* is type I error, *α*=.05, then U_*α*_=1.96. *P_1_* is the proportion of MSM in the sample, estimated to be 4% according to experts from the Chinese Center for Disease Control and Prevention and the Shenzhen Center for Disease Control and Prevention. *δ* is the tolerance error, take *δ*=0.1*P_1_*. The sample size was 9220. Assuming the refusal rate is 10%, sample size was 10,245 participants in 2014. After the presurvey, we found that the actual refusal rate was less than 10%, so in 2019, we reduced the sample size to 10,000.

### Definition and Measure

Participants who had lived in Shenzhen longer than 6 months were considered local residents, while those who had been in Shenzhen for less than 6 months were considered part of the floating population. Men who had ever had sex with another man in their lifetime were designated *MSM ever* (MSMe), and men who had sex with another man in the previous 6 months were designated *MSM active* (MSMa). Participants were asked to estimate the frequency of condom use in sex with regular male sex partners in the previous 6 months and the frequency of condom use in sex with nonregular male sex partners in the previous 6 months, with these values totaling 100%.

### Population Size of MSM Estimation Methods

The formula for estimating the population size of MSM was computed as



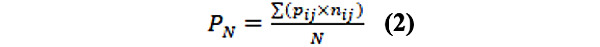



*P_N_* is the proportion of MSM in the census population of males aged 16 years and older, *p* is the proportion of MSM in participants, *n* is the population of the different age groups in the census population, *N* is the census population of males aged 16 years and older in Shenzhen, *i* is the population category (1=local, 2=floating), and *j* is the age group.

### Weighting

Considering the deviation of distribution in age and residence category (eg, local residents and floating population) between the sampled male participants and the census population of males aged 16 years and older in Shenzhen, weight adjustment was applied to control the confounding effect. The formula for calculating weight was computed as



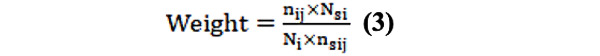



*n_s_* is the population of different age groups in the sample, *N_s_* is the population of different residence categories in the sample, *n* is the census population of different age group, *N* is the census population of male aged 16 years and older of different residence categories in Shenzhen, *i* is the residence category (1=local, 2=floating), and *j* is the age group. The distribution of MSMe and MSMa in different age groups and population categories and the final weights are shown in Multimedia Appendix 1, Table S1.

### Statistical Analysis

The raw dataset was used to estimate the population size of MSM using equation 2, and other statistical analyses were conducted with the weighted data. A chi-square test was performed to examine the difference in frequency distribution. Logistic regression analysis was used to evaluate the difference in characteristics of MSM in 2019 versus 2014. Univariate analyses were further included in the multivariate analyses with forward stepwise selection (*P*≤.05 to enter, *P*>.10 to remove). All statistical analyses were performed in SPSS (version 20, IBM Corp). Odds ratio, adjusted odds ratio, and 95% confidence intervals were presented in the results. The significance level was .05.

## Results

### Descriptive Analysis

Of the men recruited in 2014, 41.3% (4200/10,170) were aged 21 to 30 years, 48.5% (4936/10,170) were married, and 82.9% (8438/10,170) were heterosexual. A total of 49.1% (4996/10,170) of participants had a household registration in other domestic provinces or regions, 79.9% (8128/10,170) were local residents, and 45.0% (4571/10,170) had attained an educational level of college or above. Of the men recruited in 2019, 36.9% (3775/10,226) were aged between 21 and 30 years, 48.6% (4970/10,226) were married, and 86.3% (8826/10,226) were heterosexual. A total of 46.5% (4751/10,226) of participants had a household registration in other provinces or regions, 77.9% (7966/10,226) were local residents, and 52.9% (5414/10,226) had an educational level of college or above (Multimedia Appendix 2).

In general, the proportion of MSMe and MSMa varied significantly (*P*<.001) by population categories, educational levels, marital status, and sexual orientation in 2014. The proportion of MSMe varied significantly by age (*P*<.001), and the proportion of MSMa varied significantly by household registration (*P*=.02, Multimedia Appendix 2). Of the MSMe in 2014 (Table 2), 58.0% (260/448) had both male and female sex partners, 17.6% (79/448) had been diagnosed with an STI, 23.9% (107/448) had HIV test in the previous year, and 33.9% (152/448) preferred to seek sex partners in MSM venues. Among the MSMa (Table 2), 55.9% (128/229) had both male and female sex partners, 27.1% (62/229) had been diagnosed with an STI in the previous year, 37.1% (85/229) had had an HIV test in the previous year, 42.4% (97/229) reported a preference for seeking sex partner in MSM venues, 80.8% (185/229) were non-100% condom use during the previous 6 months, 70.6% (161/229) reported 2 or more sex partners, and 72.9% (167/229) reported a frequency of sex with men 2 to 4 times per month in the previous 6 months. Multivariate logistic regression analysis showed that compared to the general male population, MSMe were less educated, more likely to be floating population, have unmarried status (divorced, widowed, separation, etc), and self-identify as homosexual or bisexual. The MSMa were more likely to be floating population, have other marital status, and self-identify as homosexual or bisexual compared to the general male population (Multimedia Appendix 1, Table S2).

As shown in Multimedia Appendix 2, the proportion of MSMe in 2019 varied significantly (*P*<.05) by age, population category, educational level, household registration status, marital status, and sexual orientation. The proportion of MSMa varied significantly (*P*<.001) by population category, marital status, and sexual orientation. Among the MSMe in 2019 (Table 2), 42.4% (212/500) had both male and female sex partners, 10.2% (51/500) had been diagnosed with an STI in the previous year, and 24.6% (123/500) had an HIV test in the previous year. Among the MSMa (Table 2), 31.7% (66/208) reported that they had both male and female sex partners, 16.8% (35/208) had been diagnosed with an STI in the previous year, 40.2% (84/208) had an HIV test in the previous year, 28.4% (59/208) reported preference to seek sex partner through mobile phone apps, 57.7% (120/208) were non-100% condom use, 56.7% (118/208) reported 2 or more sex partners, and 44.2% (92/208) reported sex with men 2 to 4 times every month in the previous 6 months. The result of multivariate logistic regression analysis showed that compared to the general male population, MSMe were less likely to be unmarried, aged 16 to 20 years, and more likely to be floating population and self-identify as homosexual or bisexual. The MSMa were more likely to be floating population, in unmarried status, and self-identify as homosexual or bisexual (Multimedia Appendix 1, Table S3).

**Table 2:** Prevalence of risk behaviors among men who have ever had sex with another man (MSMe) and men who had sex with another man in the previous 6 months (MSMa) in 2014 and 2019.

		2014			2019	
		MSMe (n=448), n (%)	MSMa (n=229), n (%)	MSMe, (n=500), n (%)		MSMa (n=208), n (%)
**Gender of sex partner**						
	Male only	188 (41.96)	101 (44.10)	288 (57.60)		142 (68.27)
	Male and female	260 (58.04)	128 (55.90)	212 (42.40)		66 (31.73)
**STI^a^ history**						
	Yes	79 (17.63)	62 (27.07)	51 (10.20)		35 (16.75)
	No	369 (82.37)	167 (72.93)	449 (89.80)		174 (83.25)
**HIV testing history**						
	Yes	107 (23.88)	85 (37.12)	123 (24.60)		84 (40.19)
	No	341 (76.12)	144 (62.88)	377 (75.40)		125 (59.81)
**Preferred platform to find male sex partners**						
	Venue frequented by MSM^b^	152 (33.93)	97 (42.36)	90 (18.00)		55 (26.44)
	Internet	127 (28.35)	68 (29.69)	87 (17.40)		39 (18.75)
	Mobile phone app	71 (15.85)	39 (17.03)	119 (23.80)		59 (28.37)
	Other	98 (21.88)	25 (10.92)	197 (39.40)		55 (26.44)
**100% condom use when having sex with men**						
	No	—^c^	185 (80.79)	—		120 (57.69)
	Yes	—	44 (19.21)	—		88 (42.31)
**Number of male sex partners**						
	1	—	67 (29.4)	—		90 (43.1)
	2	—	68 (29.7)	—		40 (19.4)
	3-5	—	32 (14.1)	—		43 (20.5)
	≥6	—	61 (26.8)	—		35 (17.0)
**Frequency of sex with men**						
	≤1 time a month	—	62 (27.07)	—		74 (35.58)
	2-4 times a month	—	122 (53.28)	—		92 (44.23)
	≥2 times a week	—	45 (19.65)	—		42 (20.19)

^a^STI: sexually transmitted infections.

^b^MSM: men who have sex with men.

^c^Not applicable.

### Estimating the Population Size of MSM

In 2014, 448 (4.41%, 95% CI 4.01%-4.80%) participants were MSMe, and 229 (2.25%, 95% CI 1.96%-2.54%) were MSMa. In 2019, 500 (4.90%, 95% CI 4.47%-5.31%) were MSMe, and 208 (2.03%, 95% CI 1.76%-2.31%) participants were MSMa. Proportions of MSMe (*P*=.10) and MSMa (*P*=.28) were not significantly different between 2014 and 2019.

The proportion of MSMe in the male population aged 16 years and older was 4.51% (305,984/6,782,813, 95% CI 4.50%-4.53%) and 4.91% (400,689/8,158,157, 95% CI 4.90%-4.93%) in Shenzhen in 2014 and 2019, respectively. The proportion of MSMa in the male population aged 16 years and older was 2.29% (155,469/6,782,813, 95% CI 2.28%-2.30%) and 2.05% (167,337/8,158,157, 95% CI 2.04%-2.06%) in Shenzhen in 2014 and 2019, respectively.

In 2019, there were about 587.8 million males aged 16 years and older in China. Based on the proportion of MSM in Shenzhen in 2019, we estimated that the MSMe and MSMa population sizes in China were 30,434,062 (5.72%, 95% CI 5.72%-5.72%) and 12,005,445 (2.04%, 95% CI 2.04%-2.04%), respectively.

### Change in Characteristics of MSMe

**Figure 2:**
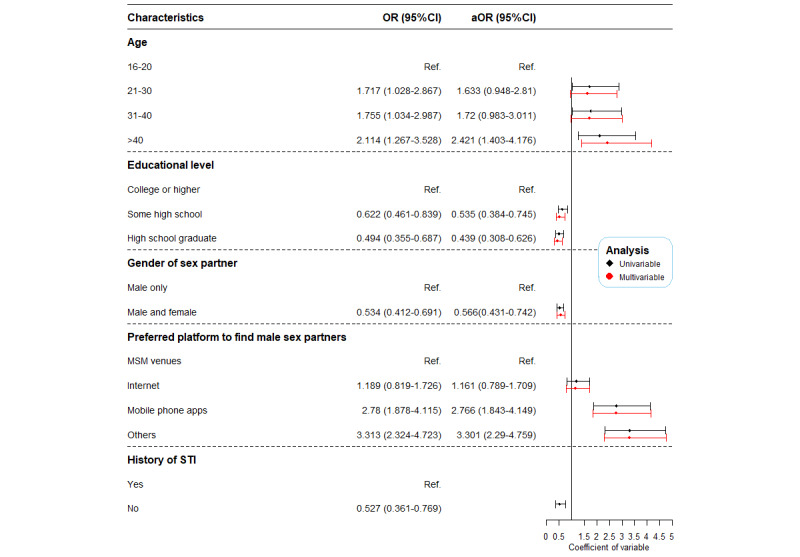
Changes in characteristics among men who have ever had sex with another man—2019 versus 2014. OR: odds ratio; ref: reference; MSM: men who have sex with men; STI: sexually transmitted disease.

Figure 2 summarized the difference in the characteristics of MSMe in 2014 and 2019. Based on the results of univariate logistic regression analysis, the variables age, length of stay in Shenzhen, educational level, gender of sex partner, preferred ways of seeking sex partners, and having been diagnosed with an STI in the previous year (*P*<.05) were included in multivariate logistic regression analysis. Compared to MSMe in 2014, MSMe in 2019 were more likely to be aged 41 years and older (aOR 2.42, 95% CI 1.40-4.18), preferred to seek sex partners through mobile phone apps (aOR 2.77, 95% CI 1.84-4.15) or other ways (aOR 3.30, 95% CI 2.29-4.76),were less likely to have an educational level of high school (aOR 0.44, 95% CI 0.31-0.63) or junior middle school or below (aOR 0.54, 95% CI 0.38-0.76), and reported having both male and female sex partners (aOR 0.57, 95% CI 0.43-0.74).

### Change in Characteristics of MSMa

**Figure 3:**
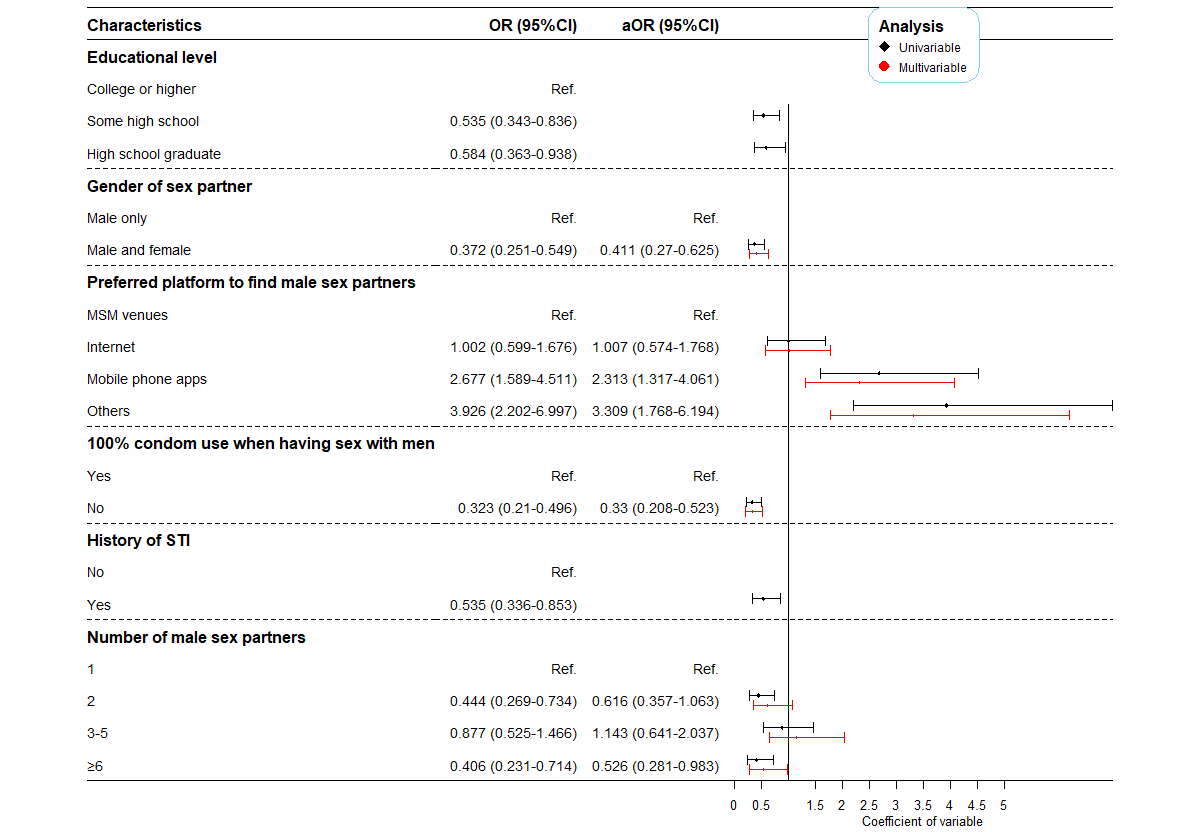
Changes in characteristics among men who had sex with another man in the previous 6 months—2019 versus 2014. OR: odds ratio; ref: reference; MSM: men who have sex with men; STI: sexually transmitted disease.

Figure 3 summarized the difference in characteristics of MSMa in 2014 and 2019. Variables including educational levels, gender of sex partner, non-100% condom use in the previous 6 months, being diagnosed with an STI in the previous year, preferred ways of social networking, and number of sex partners in the previous 6 months (*P*<.05) were added in the multivariate logistic regression model based on the results of univariate logistic regression analysis. The result showed that in 2019, MSMa were more likely to seek sex partner through mobile phone apps (aOR 2.31, 95% CI 1.32-4.06) and were less likely to have male and female sex partners (aOR 0.41, 95% CI 0.27-0.63), non-100% condom use (aOR 0.33, 95% CI 0.21-0.52), and more than 6 sex partners reported in the previous 6 months (aOR 0.53, 95% CI 0.28-0.98) compared to MSMa in 2014.

## Discussion

### Principal Findings

Accurate estimation of the population size of MSM is of great importance to develop HIV prevention and intervention and the strategy to achieve the 90-90-90 goals. As far as we know, this is the first study to estimate the MSM population size by population survey with random sampling in China. Based on this study, the population size of MSMa was estimated to be 154,059 in 2014 and 166,464 in 2019 in Shenzhen, higher than the 70,000 MSM estimated to be in Shenzhen in 2014 [20]; the population of MSMa in China was 12,005,445 in 2019, and the population of MSM in China was 8,288,536 in 2018 [9]. However, previous studies estimating the population size of MSM were based on the MSM venues [20] or social media [9] by capture-recapture, which recruited the sample from a specific group of MSM and might not fully represent the whole MSM population. In addition, the estimation of MSM population size based on social media may result in a high risk of bias because of duplicate registration in the app. For example, in Blued, the largest homosexual social media in China, the actual population size of MSM could be lower than the number of monthly active user (12 million in 2016) due to duplicate registration [22]. Furthermore, a previous study based on this social media only recruited Blued users as MSM, which might ignore the MSM who did not use this app and underestimate the population size of MSM in China [9]. In this study, we used a population survey with a large sample size of the general population to recruit MSM. It represented the whole MSM population and reduced the bias of estimation. Therefore, the results of this study could be reliable to reflect the actual population size of MSM.

The higher proportion of MSMe in 2019 compared to that in 2014 could mainly be attributed to the cumulative effect of time. Unlike MSMe, MSMa were sexually active and the main risky population in HIV prevention and control [23]. Since the reform and opening-up policy completed in the 1980s in China, the concept of sex among Chinese people has changed [24]. Casual sexual behavior has become more acceptable [25]. However, due to the lack of sex education, some teenagers learn about sexual orientation on their own, which may lead them to be induced to be homosexual [26,27]. The Chinese government has paid more attention to sex education since 2015 [28]. Teenagers can acquire sexual knowledge from school since 2015, and the whole society has become tolerant of homosexual behavior [29]. Consequently, teenagers can through the correct channels to learn sexual knowledge, including sexual orientation, which led to the proportion of MSMa being lower in 2019 than it was in 2014. Additionally, the prevalence of bisexual behaviors among MSMa was lower in 2019 than it was in 2014. With increasing tolerance of homosexuals in society [30], MSM would no longer have to get married to avoid stigma and social pressure. Therefore, the proportion of bisexual behavior was decreased accordingly, which helps reduce the risk of HIV transmission from MSM to women.

The epidemic of HIV/AIDS has been getting worse in China in the past decade [31] but declined in Shenzhen since 2017. This might be attributed to China’s HIV treatment strategies and the reduced prevalence of HIV risk behavior among MSM in Shenzhen. China’s HIV treatment strategy includes universal access to HIV Voluntary Counseling and Testing Clinics and free HIV treatment, which allows for greater access to testing and higher treatment adherence for people living with HIV in China than in other countries, thus facilitating our ability to reach the Undetectable=Untransmittable goal and even the 2030 goal of ending AIDS [32,33]. Shenzhen Center for Disease Control and Prevention has applied cross-sectional surveys on MSM annually by collecting samples in MSM venues or through social networks of these venue-based MSM since 2008 [34] and has implemented venue-specific interventions covering all the MSM venues in Shenzhen since 2015 [35]. The results of surveillance on MSM suggested that the prevalence of HIV risk behavior among the MSM who prefer to seek sex partners in specific venues was reduced after implementation of the venue-specific intervention [36]. However, because the series of cross-sectional studies only covered venue-based MSM and other MSM within their social network, the results cannot be extrapolated to the whole MSM group in Shenzhen. In this study, we found that prevalence of non-100% condom use, bisexual behavior, and more than 5 sex partners in the previous 6 months among MSMa in 2019 were much lower than that in 2014 and lower than in other countries [37]. In addition, the prevalence of HIV risk behavior was significantly lower in 2019 than it was in 2014 for MSM who preferred to seek sex partners in venues and other MSM subgroups. This finding indicates that the venue-specific interventions can not only directly influence MSM who preferred to seek sex partners in venues but also indirectly influence the whole population of MSM. The results of continuous surveillance based on MSM venues could help to deduce HIV risk behavior changes in the whole MSM population.

Data from annual MSM surveillance and this study suggested that the preferred way of seeking sex partners among MSMa changed from MSM venue to mobile phone apps from 2014 to 2019. A study in the United States suggested that gay app users had a higher risk of HIV infection and reported more sex partners and non-100% condom use than nonusers [38]. However, our study found that the prevalence of multiple sex partners among MSM who preferred to seek sex partners in venues was higher than other MSM, consistent with a series of cross-sectional studies [36] indicating that in Shenzhen, MSM who preferred to seek sex partners in venues have a higher risk than others of HIV infection. Furthermore, this study showed that 26.4% of MSMa sought sex partners in other ways (other-than-MSM venues, internet, and mobile phone apps) in 2019, increased by 3.3 times compared to that in 2014. That means MSM social networking preferences have become more diverse and private, leading to the increase of hidden MSM. This finding further verified that samples only from MSM venues, MSM websites, or social media might have selection bias and limit the representativeness of the whole MSM population.

In this study, we also found weak positive correlation between the prevalence of diagnosed STIs in the previous year and the prevalence of non-100% condom use among MSMa. The prevalence of diagnosed STIs in the previous year among MSMa was significantly decreased in univariate analysis from 2014 to 2019. However, the difference was not significant in the multivariate analysis; this may be attributed to the collinearity between the prevalence of diagnosed STIs in the previous year and the prevalence of non-100% condom use. Nevertheless, the causal relationships need to be validated in future studies.

### Limitations

Our study had several limitations. First, the result only reflected the population of Shenzhen, which might not be generalizable to the whole country. However, we have controlled the confounders and made a weight adjustment according to the census population, so the results of this study could offer a reference for other cities or areas in China. Second, MSM might conceal having sex with men because of the stigma and therefore induce reporting bias. We did our best to allow for this possibility by using self-administered questionnaires to elicit self-reported behavior and beliefs, which may have reduced the underreporting of sensitive behavior. All participants were informed that their contents were not visible to the field investigators and the questionnaire would not collect any personal identifiable information, which could prompt respondents to complete the questionnaire truthfully. Third, because of the sensitivity of the survey content, this study had a certain rate of rejection. Still, we calculated the population size by weighting on age and residence status. We also roughly assessed the age distribution of respondents and nonrespondents and found that they are similar.

### Conclusions

In Shenzhen, the proportion of MSMa among the general male population in 2019 was lower than in 2014, and the prevalence of HIV risk behavior was reduced in 2019. Although the preferred platform to find male sex partners among MSM has changed, the intervention on high HIV risk MSM could still help to reduce HIV risk behaviors among the whole MSM group. And because the MSM preferred to seek sex partners through mobile phone apps, further study is needed to strengthen internet intervention on high HIV risk MSM to curb the spread of HIV.

## Appendix

Multimedia Appendix 1 Supplementary tables.

Multimedia Appendix 2 Proportion of men who have ever had sex with another man and men who had sex with another man in the previous 6 months among men in Shenzhen in 2014 and 2019 stratified by sociodemographic characteristics.

## Abbreviations

aOR: adjusted odds ratio
MSM: men who have sex with men
MSMa: men who had sex with another man in the previous 6 months
MSMe: men who have ever had sex with another man
OR: odds ratio
STI: sexually transmitted infection

## References

[1] Beyrer C, Baral SD, Collins C, Richardson ET, Sullivan PS, Sanchez J, Trapence G, Katabira E, Kazatchkine M, Ryan O, Wirtz AL, Mayer KH (2016). The global response to HIV in men who have sex with men. Lancet.

[2] Tao J, Vermund SH, Lu H, Ruan Y, Shepherd BE, Kipp AM, Amico KR, Zhang X, Shao Y, Qian H (2017). Impact of depression and anxiety on initiation of antiretroviral therapy among men who have sex with men with newly diagnosed HIV infections in China. AIDS Patient Care STDS.

[3] The Joint United Nations Programme on HIV/AIDS National HIV sentinel surveillance.

[4] STD and AIDS Prevention and Control Center (2012). The epidemic situation of AIDS and STD in China and the progress of major prevention and treatment work in 2011. Chinese J AIDS STD.

[5] National Health Commission of the People's Republic of China (2019). New progress in AIDS prevention and control in China in 2019. Chinese J AIDS STD.

[6] The proportion of menstrual transmission among new AIDS cases in Shenzhen is 97%.

[7] Nguyen P, Gilmour S, Le P, Onishi K, Kato K (2021). Progress toward HIV elimination goals: trends in and projections of annual HIV testing and condom use in Africa. AIDS.

[8] (2013). UNAIDS: guidelines on estimating the size of populations most at risk to HIV.

[9] Hu M, Xu C, Wang J (2020). Spatiotemporal analysis of men who have sex with men in mainland china: social app capture-recapture method. JMIR Mhealth Uhealth.

[10] Jing L, Cui Y, Lu Q, Yu H (2020). Multiplier method estimates of the population of men who have sex with men: the effect of privacy protection. J Public Health (Oxf).

[11] Guo W, Bao S, Lin W, Wu G, Zhang W, Hladik W, Abdul-Quader A, Bulterys M, Fuller S, Wang L (2013). Estimating the size of HIV key affected populations in Chongqing, China, using the network scale-up method. PLoS One.

[12] Marcus U, Hickson F, Weatherburn P, Schmidt AJ, EMIS Network (2013). Estimating the size of the MSM populations for 38 European countries by calculating the survey-surveillance discrepancies (SSD) between self-reported new HIV diagnoses from the European MSM internet survey (EMIS) and surveillance-reported HIV diagnoses among MSM in 2009. BMC Public Health.

[13] Wang Y, Hu Z, Peng K, Xin Y, Yang Y, Drescher J, Chen R (2019). Discrimination against LGBT populations in China. Lancet Public Health.

[14] Ding C, Chen X, Wang W, Yu B, Yang H, Li X, Deng S, Yan H, Li S (2020). Sexual minority stigma, sexual orientation concealment, social support and depressive symptoms among men who have sex with men in China: a moderated mediation modeling analysis. AIDS Behav.

[15] Zou H, Fan S (2017). Characteristics of men who have sex with men who use smartphone geosocial networking applications and implications for HIV interventions: a systematic review and meta-analysis. Arch Sex Behav.

[16] Wang Z, Yang X, Mo PKH, Fang Y, Ip TKM, Lau JTF (2020). Influence of social media on sexualized drug use and chemsex among Chinese men who have sex with men: observational prospective cohort study. J Med Internet Res.

[17] Zhu Z, Yan H, Wu S, Xu Y, Xu W, Liu L, Li X, Xu F, Detels R (2019). Trends in HIV prevalence and risk behaviours among men who have sex with men from 2013 to 2017 in Nanjing, China: a consecutive cross-sectional survey. BMJ Open.

[18] Dong M, Peng B, Liu Z, Ye Q, Liu H, Lu X, Zhang B, Chen J (2019). The prevalence of HIV among MSM in China: a large-scale systematic analysis. BMC Infect Dis.

[19] Mercer C, Fenton K, Copas A, Wellings K, Erens B, McManus S, Nanchahal K, Macdowall W, Johnson AM (2004). Increasing prevalence of male homosexual partnerships and practices in Britain 1990-2000: evidence from national probability surveys. AIDS.

[20] Zhao J, Chen L, Cai W, Tan J, Tan W, Zheng C, Cheng J, Yang Z, He M, Wang X (2014). HIV infection and sexual behaviors among non-commercial men who have sex with men at different venues. Arch Sex Behav.

[21] Zhao J, Cai W, Zheng C, Yang Z, Xin R, Li G, Wang X, Chen L, Zhong P, Zhang C (2014). Origin and outbreak of HIV-1 CRF55_01B among MSM in Shenzhen, China. J Acquir Immune Defic Syndr.

[22] Wang L, Podson D, Chen Z, Lu H, Wang V, Shepard C, Williams JK, Mi G (2019). Using social media to increase HIV testing among men who have sex with men—Beijing, China, 2013-2017. MMWR Morb Mortal Wkly Rep.

[23] Prestage GP, Hudson J, Down I, Bradley J, Corrigan N, Hurley M, Grulich AE, McInnes D (2009). Gay men who engage in group sex are at increased risk of HIV infection and onward transmission. AIDS Behav.

[24] Yu J (2012). Teenage sexual attitudes and behaviour in China: a literature review. Health Soc Care Community.

[25] Li G, Jiang Y, Zhang L (2019). HIV upsurge in China's students. Science.

[26] Hébert M, Cénat JM, Blais M, Lavoie F, Guerrier M (2016). Child sexual abuse, bullying, cyberbullying, and mental health problems among high schools students: a moderated mediated model. Depress Anxiety.

[27] Young JC, Widom CS (2014). Long-term effects of child abuse and neglect on emotion processing in adulthood. Child Abuse Negl.

[28] Burki T (2016). Sex education in China leaves young vulnerable to infection. Lancet Infect Dis.

[29] Tao J, Ruan Y, Yin L, Vermund SH, Shepherd BE, Shao Y, Qian H (2013). Sex with women among men who have sex with men in China: prevalence and sexual practices. AIDS Patient Care STDS.

[30] Chua RYJ, Huang KG, Jin M (2019). Mapping cultural tightness and its links to innovation, urbanization, and happiness across 31 provinces in China. Proc Natl Acad Sci U S A.

[31] National Health Commission of the People's Republic of China (2019). Strategy and analysis of AIDS prevention and control in China.

[32] Le PM, Nguyen PT, Nguyen HV, Bui DH, Vo SH, Nguyen NV, Nguyen TV, Tran AT, Le AD, Ha NM, Dao AT, Gilmour S (2021). Adherence to highly active antiretroviral therapy among people living with HIV and associated high-risk behaviours and clinical characteristics: a cross-sectional survey in Vietnam. Int J STD AIDS.

[33] Nguyen PT, Rahman MS, Le PM, Nguyen HV, Vu KD, Nguyen HL, Dao ATM, Khuong LQ, Hoang MV, Gilmour S (2021). Trends in, projections of, and inequalities in reproductive, maternal, newborn and child health service coverage in Vietnam 2000-2030: a Bayesian analysis at national and sub-national levels. Lancet Reg Health West Pac.

[34] Huang Y, Zhang Y, Li K, Zhao J (2016). Changes in prevalence of HIV or syphilis among male sex workers and non-commercial men who have sex with men in Shenzhen, China: results of a second survey. PLoS One.

[35] Tan JG, Cheng JQ, Lu ZX (2012). [Evaluation of effects of combination intervention model to men who have sex with men]. Chin J Prev Med.

[36] Wei L, Chen L, Zhang H, Yang Z, Zou H, Yuan T, Xiao Y, Liu S, Tan W, Xie W, Liu L, Cheng J, Zhao J (2019). Use of gay app and the associated HIV/syphilis risk among non-commercial men who have sex with men in Shenzhen, China: a serial cross-sectional study. Sex Transm Infect.

[37] Nguyen PT, Gilmour S, Le PM, Nguyen TT, Tanuma J, Nguyen HV (2021). Factors associated with high-risk behaviors of people newly diagnosed with HIV/AIDS: results from a cross-sectional study in Vietnam. AIDS Care.

[38] Abuelezam NN, Reshef YA, Novak D, Grad YH, Seage Iii GR, Mayer K, Lipsitch M (2019). Interaction patterns of men who have sex with men on a geosocial networking mobile app in seven United States metropolitan areas: observational study. J Med Internet Res.

