# Supplementary material for: Estimating Changes in Population Size and Behavioral Characteristics in Men Who Have Sex With Men Between 2014 and 2019: Longitudinal Study
Source: JMIR Public Health Surveill. 2022 Aug 16;8(8):e34150. doi: 10.2196/34150 (PMC9428784; doi:10.2196/34150)
Supplement: Multimedia Appendix 1 [file publichealth_v8i8e34150_app1.docx]

Multimedia Appendix 1. Supplementary tables.

**Table S1.** Weights and distribution of MSM in different age groups and population categories.

|  | floating population n(%) | | | | | local residents n(%) | | | | |
| --- | --- | --- | --- | --- | --- | --- | --- | --- | --- | --- |
|  | MSMe | | MSMa | | **weights** | MSMe | | MSMa | | **weights** |
|  | No | Yes | No | Yes |  | No | Yes | No | Yes |  |
| **2014** |  |  |  |  |  |  |  |  |  |  |
| 16-20 | 211(91.7) | 19(8.3) | 220(95.7) | 10(4.3) | 0.5073 | 731(96.3) | 28(3.7) | 745(98.2) | 14(1.8) | 0.8415 |
| 21-30 | 827(94.5) | 48(5.5) | 849(97) | 26(3.0) | 0.7543 | 3222(96.9) | 103(3.1) | 3267(98.3) | 58(1.7) | 0.7339 |
| 31-40 | 507(91.7) | 46(8.3) | 528(95.5) | 25(4.5) | 2.3167 | 2104(97) | 65(3) | 2139(98.6) | 30(1.4) | 1.3028 |
| 41-50 | 241(90.6) | 25(9.4) | 255(95.9) | 11(4.1) | 2.7690 | 1223(94.9) | 66(5.1) | 1255(97.4) | 34(2.6) | 1.8727 |
| 51-60 | 78(95.1) | 4(4.9) | 79(97.5) | 2(2.5) | 1.1747 | 347(94) | 22(6) | 363(98.4) | 6(1.6) | 1.4874 |
| >60 | 28(75.7) | 9(24.3) | 34(91.9) | 3(8.1) | 1.4650 | 204(93.6) | 14(6.4) | 208(95.9) | 9(4.1) | 2.3130 |
| **2019** |  |  |  |  |  |  |  |  |  |  |
| 16-20 | 243(95.7) | 11(4.3) | 251(98.4) | 4(1.6) | 0.3628 | 609(97.1) | 18(2.9) | 622(99) | 6(1) | 0.6333 |
| 21-30 | 923(95.3) | 46(4.7) | 947(97.7) | 22(2.3) | 0.8356 | 2689(95.8) | 117(4.2) | 2744(97.8) | 62(2.2) | 0.7800 |
| 31-40 | 574(93.6) | 39(6.4) | 594(96.9) | 19(3.1) | 2.3125 | 2034(96) | 84(4) | 2081(98.3) | 37(1.7) | 1.0080 |
| 41-50 | 261(88.5) | 34(11.5) | 276(93.6) | 19(6.4) | 3.7241 | 1293(93.6) | 88(6.4) | 1360(98.5) | 21(1.5) | 1.7220 |
| 51-60 | 74(82.2) | 16(17.8) | 86(96.6) | 3(3.4) | 3.2037 | 588(95.9) | 25(4.1) | 608(99.2) | 5(0.8) | 2.7492 |
| >60 | 35(85.4) | 6(14.6) | 38(92.7) | 3(7.3) | 1.4476 | 403(96) | 17(4) | 412(98.1) | 8(1.9) | 1.6742 |

**Table S2.** Multivariate logistic regressions of demographic distribution among MSMe and MSMa in 2014.

|  | **MSMe** | | **MSMa** | |
| --- | --- | --- | --- | --- |
|  | Adjusted odds ratio | 95% confidence intervals | Adjusted odds ratio | 95% confidence intervals |
| **Population category** |  |  |  |  |
| Floating population | **1.644** | **1.312-2.059** | **1.662** | **1.239-2.331** |
| Local residents | ref. |  | ref. |  |
| **Educational levels** |  |  |  |  |
| Some high school | **1.733** | **1.347-2.229** |  |  |
| High school graduate | **1.408** | **1.089-1.821** |  |  |
| College or higher | ref. |  |  |  |
| **Sexual orientation** |  |  |  |  |
| Bisexual | **15.607** | **11.935-20.408** | **19.533** | **13.295-28.700** |
| Homosexual | **18.528** | **14.486-24.698** | **23.336** | **16.347-33.312** |
| Heterosexual | ref. |  | ref. |  |
| **Marital status** |  |  |  |  |
| Unmarried | ref. |  | ref. |  |
| Married | 0.913 | 0.733-1.138 | 0.814 | 0.606-1.095 |
| Others | **2.110** | **1.486-2.995** | **2.277** | **1.479-3.504** |

**Table S3.** Multivariate logistic regressions of demographic distribution among MSMe and MSMa in 2019.

|  | **MSMe** | | **MSMa** | |
| --- | --- | --- | --- | --- |
|  | Adjusted odds ratio | 95% confidence intervals | Adjusted odds ratio | 95% confidence intervals |
| **Age** |  |  |  |  |
| 16-20 | **0.469** | **0.287-0.767** |  |  |
| 21-30 | **0.724** | **0.528-0.994** |  |  |
| 31-40 | **0.725** | **0.552-0.951** |  |  |
| >40 | ref. |  |  |  |
| **Population category** |  |  |  |  |
| Floating population | **1.416** | **1.131-1.772** | **1.443** | **1.053-1.978** |
| Local residents | ref. |  | ref. |  |
| **Sexual orientation** |  |  |  |  |
| Bisexual | **23.621** | **18.227-30.612** | **37.450** | **23.526-59.615** |
| Homosexual | **40.386** | **31.390-51.961** | **70.902** | **45.458-110.586** |
| Heterosexual | ref. |  | ref. |  |
| **Marital status** |  |  |  |  |
| Unmarried | **ref.** |  | **ref.** |  |
| Married | **0.730** | **0.552-0.966** | **0.654** | **0.478-0.896** |
| Others | 1.514 | 0.995-2.305 | **2.093** | **1.288-3.399** |
