# Supplementary material for: Estimating Changes in Population Size and Behavioral Characteristics in Men Who Have Sex With Men Between 2014 and 2019: Longitudinal Study
Source: JMIR Public Health Surveill. 2022 Aug 16;8(8):e34150. doi: 10.2196/34150 (PMC9428784; doi:10.2196/34150)
Supplement: Multimedia Appendix 2 [file publichealth_v8i8e34150_app2.docx]

Multimedia Appendix 2. Proportion of men who have ever had sex with another man (MSMe) and men who had sex with another man (MSMa) among men in Shenzhen 2014 and 2019 stratified by sociodemographic characteristics.

|  | 2014 (sample size=10170) | | | | | 2019 (sample size=10226) | | | | | Total for two years |
| --- | --- | --- | --- | --- | --- | --- | --- | --- | --- | --- | --- |
|  | MSMe | | MSMa | | Total (N_1_) | MSMe | | MSMa | | Total (N_2_) |  |
|  | n (%) | *P* value^a^ | n (%) | *P* value |  | n (%) | *P* value | n (%) | *P* value |  |  |
| **Age** |  | <0.001 |  | 0.108 |  |  | <0.001 |  | 0.153 |  |  |
| 16-20 | 47(4.75) |  | 24(2.43) |  | 989 | 29(3.29) |  | 9(1.02) |  | 882 | 1871 |
| 21-30 | 151(3.6) |  | 84(2) |  | 4200 | 163(4.32) |  | 84(2.23) |  | 3775 | 7975 |
| 31-40 | 111(4.08) |  | 55(2.02) |  | 2722 | 123(4.5) |  | 56(2.05) |  | 2731 | 5453 |
| >40 | 139(6.16) |  | 65(2.88) |  | 2258 | 185(6.52) |  | 59(2.08) |  | 2839 | 5097 |
| **Population category** |  | <0.001 |  | <0.001 |  |  | <0.001 |  | <0.001 |  |  |
| Floating population | 150(7.34) |  | 78(3.82) |  | 2042 | 151(6.68) |  | 69(3.05) |  | 2260 | 4302 |
| Local residents | 298(3.67) |  | 151(1.86) |  | 8128 | 348(4.36) |  | 140(1.75) |  | 7966 | 16094 |
| **Educational level** |  | <0.001 |  | <0.001 |  |  | <0.001 |  | 0.298 |  |  |
| Some high school | 172(7.09) |  | 82(3.38) |  | 2427 | 171(7.46) |  | 56(2.44) |  | 2292 | 4719 |
| High school graduate | 138(4.35) |  | 64(2.02) |  | 3172 | 108(4.28) |  | 47(1.86) |  | 2521 | 5693 |
| College or higher | 138(3.02) |  | 83(1.82) |  | 4571 | 220(4.06) |  | 106(1.96) |  | 5414 | 9985 |
| **Household registration** |  | 0.069 |  | 0.015 |  |  | 0.037 |  | 0.902 |  |  |
| Shenzhen | 144(5.14) |  | 79(2.82) |  | 2799 | 182(5.7) |  | 67(2.1) |  | 3192 | 5991 |
| Other cities in Guangdong | 104(4.38) |  | 58(2.44) |  | 2376 | 105(4.6) |  | 43(1.89) |  | 2281 | 4657 |
| Other provinces | 201(4.02) |  | 91(1.82) |  | 4996 | 213(4.48) |  | 95(2) |  | 4751 | 9747 |
| **Marital status** |  | <0.001 |  | <0.001 |  |  | <0.001 |  | <0.001 |  |  |
| Unmarried | 189(4.05) |  | 103(2.21) |  | 4670 | 229(4.81) |  | 106(2.23) |  | 4764 | 9434 |
| Married | 204(4.13) |  | 93(1.88) |  | 4934 | 223(4.49) |  | 76(1.53) |  | 4970 | 9904 |
| Others | 47(4.75) |  | 33(2.43) |  | 565 | 47(9.53) |  | 27(5.48) |  | 493 | 1058 |
| **Sexual orientation** |  | <0.001 |  | <0.001 |  |  | <0.001 |  | <0.001 |  |  |
| Bisexual | 136(18.21) |  | 72(9.64) |  | 747 | 168(22.83) |  | 71(9.65) |  | 736 | 1483 |
| Homosexual | 203(20.59) |  | 113(11.46) |  | 986 | 226(34.04) |  | 112(16.87) |  | 664 | 1650 |
| Heterosexual | 109(1.29) |  | 44(0.52) |  | 8438 | 106(1.2) |  | 25(0.28) |  | 8826 | 17264 |

^a^Chi-square test was performed to examine the difference in frequency distribution, and the significance level was *P*<.05.
